# Characterization and comparative genomic analysis of a novel bacteriophage against an extremely drug-resistant *Pseudomonas aeruginosa* ST1971

**DOI:** 10.1128/spectrum.02713-25

**Published:** 2026-05-21

**Authors:** Jie Jiang, Li Zhao, Chongzhi Cai, Liuyang Hu, Shuping Qin, Liang Liang, Yulin Yuan

**Affiliations:** 1 Department of Clinical Laboratory, the People’s Hospital of Guangxi Zhuang Autonomous Region (Guangxi Academy of Medical Science), Nanning, Guangxi, China; Institut National de Santé Publique du Québec, Sainte-Anne-de-Bellevue, Québec, Canada

**Keywords:** multidrug-resistant, *Pseudomonas aeruginosa*, ST1971, phage

## Abstract

Given the rising prevalence of multidrug-resistant strains, *Pseudomonas aeruginosa* necessitates alternative therapies, and phages are emerging as promising antibiotic alternatives. Our study introduces a novel lytic phage, CRPAP1904, exhibiting potent activity against an extremely drug-resistant *P. aeruginosa* ST1971 strain CRPA190. Phylogenetic and comparative genomic analyses validated the novelty of phage CRPAP1904, suggesting that it represents a new species of the *Kochitakasuvirus* genus. The phage CRPAP1904 has a double-stranded DNA genome of 62,430 bp base pairs, a GC content of 60.4% and predicted 83 open reading frames. Notably, no genes associated with virulence or antibiotic resistance were detected, suggesting its suitability for therapeutic applications. We further assessed the phage’s host range, optimal multiplicity of infection (MOI), one-step growth curves, physicochemical stability, and antibacterial activity, which are critical for its application in environmental and clinical settings. The phage exhibits a broad host range, lysing 83% (20/24) of tested strains. The optimal MOI was 0.01 with a large burst size of 109 PFU/cell. Additionally, the phage exhibited stability over a pH range of 6–8 and at temperatures ranging from −20°C to 60°C. *In vitro*, the phage significantly suppresses host bacterial growth at an MOI of 100 for the first 12 h, although resistant mutants gradually emerge during prolonged exposure. In conclusion, the newly discovered phage CRPAP1904 expands phage resources and supports medicinal phage library development, advancing phage therapy and biocontrol applications in MDR-PA infections.

**IMPORTANCE:**

This study isolated a novel phage, CRPAP1904, from the hospital's sewage that specifically targets an extremely drug-resistant *Pseudomonas aeruginosa* ST1971 strain CRPA190. Genomic analysis identified CRPAP1904 as a new Kochitakasuvirus member lacking antibiotic resistance genes and virulence factors. CRPAP1904 shows excellent stability and lytic activity against MDR-PA clinical isolates. As the first phage against *Pseudomonas aeruginosa* ST1971, this work expands phage resources and supports medicinal phage library development, advancing phage therapy and biocontrol applications in MDR-PA infections.

## INTRODUCTION

*Pseudomonas aeruginosa (P. aeruginosa),* as a common gram-negative opportunistic pathogen*,* is widely distributed in natural environments such as air, water, and soil. It was designated by the World Health Organization (WHO) as a high-priority pathogen in 2024 (1). *P. aeruginosa* can cause a wide variety of acute and chronic human infections, including in patients with severe burn wounds, AIDS, urinary tract infections, chronic obstructive pulmonary disease, lung cancer, bronchiectasis, and cystic fibrosis (2, 3).

In recent decades, broad-spectrum antibiotics have been overused, leading to the development of drug resistance in *P. aeruginosa*. The rapid emergence of multidrug-resistant *P. aeruginosa* (MDR-PA) is a serious public health threat (4). They are difficult to treat due to limited treatment options and therefore are associated with serious infections with high mortality rates (5, 6). Among them, ST1971 *P. aeruginosa* strain is known to circulate in China. It expresses high adaptability to various environmental conditions and has the potential for widespread dissemination, which was identified as a highly virulent and extremely drug-resistant clone (7–12). Currently, the ST1971 clone of *P. aeruginosa* is not only endemic in China but also has been isolated in Greece (7). As an extremely drug-resistant clone, ST1971 *P. aeruginosa* strain is difficult to treat. Specifically, as the first blaNDM-1 ST1971 Chinese strain, CRPA190 was almost resistant to all common antibiotics including carbapenem, polymyxin B, and ceftazidime-avibactam (10).

Phages are natural predators of bacteria. Recently, compassionate experimental phage therapy has shown promise in targeting life-threatening MDR infections due to its long-standing historical use, apparent lack of adverse effects, and solid support by fundamental research (13, 14). In the environment, there are vast expanses of uncharacterized phages, strongly suggesting that novel phages with more desirable therapeutic properties are waiting to be discovered. The phage biobanks as potential tools to rapidly and cost-effectively manage antimicrobial resistance in the developing world have already shown their utility in providing a valuable resource to screen for therapeutic phage (15).

This study aims to characterize a newly isolated lytic bacteriophage, CRPAP 1904, assess its efficacy against ST1971 type strain CRPA190, and explore its genomic features for potential therapeutic application. Detailed characterization and comprehensive bioinformatic analysis of the bacteriophage were performed. Our findings will not only enrich the phage library but also offer a theoretical foundation and practical strategies for controlling MDR-PA infections in hospitals, as well as facilitating the development of phage-based biocontrol formulations against this pathogen.

## MATERIALS AND METHODS

### Bacterial strains and growth conditions

The isolate of carbapenem-resistant *Pseudomonas aeruginosa* ST1971 strain CRPA190, previously isolated and identified from a patient with severe pneumonia, was used for phage isolation and identification (10). It was stored at −80°C in 15% (vol/vol) glycerol and routinely grown on Luria-Bertani (LB) agar and in LB broth at 37°C. All of the bacterial strains used in this study are listed in Table S1.

### Phage isolation and purification

Phage isolation and purification were performed as described previously with minor modifications (16, 17). Briefly, sewage sample from the People’s Hospital of Guangxi Zhuang Autonomous Region was centrifuged at 8,000 × *g* for 5 min, and the supernatant was mixed with equal volumes of a mid-log phase CRPA190 culture. The mixture was incubated for 48 h at 37°C with shaking, then centrifuged at 5,000 × *g* for 5 min to pellet cells and debris. The supernatant was filtered through a 0.22 μm filter, and the filtrate was serially diluted, mixed with host strains CRPA190 in molten semisolid soft agar (0.75% wt/vol agar), and poured onto solidified 1.5% wt/vol LB agar plates. After solidification, all overlay agar plates were incubated overnight at 37°C to assess plaque formation. Subsequently, this step was repeated at least three times until plaques of consistent appearance and size were obtained, indicating phage purification. The double-layer agar plaque assay was used to determine the phage titer (calculated as the number of plaques on double-layer agar plates × dilution factor × 10) and the purified high-titer phage solutions were stored in SM (100 mM NaCl, 8 mM MgSO4⋅7H_2_O, and 50 mM Tris-HCl at pH 7.5) buffer at 4°C (18).

### Transmission electron microscopy (TEM)

Transmission electron microscopy (TEM) was conducted to measure the morphology of purified phage. A 10 μL aliquot of the purified high-titer phages (10^10^ PFU/mL) was loaded onto a copper grid and incubated for 1 min. Next, the negative staining with 2% uranyl acetate was carried out. The stained grid was subjected to TEM analysis (TECNAI G2 F20 S-Twin, USA) at an acceleration voltage of 80 kV.

### Host range analysis

The host range of the phage was determined based on 24 clinical MDR-PA strains using Plaque dot assay with minor modifications (19). All of the bacterial strains are listed in Table S1. Briefly, 100 μL of the 24 tested strains was added to 5 mL of soft 0.75% LB agar and poured onto 1.5% LB agar plates, respectively. After solidification, 10 μL of purified phage suspension (10^10^ PFU/mL) was spotted onto double-layer agar plates. After adsorption of phage suspension, plates were incubated overnight at 37°C. The formation of plaques on the bacterial lawns of the tested strains was observed. To strengthen the host range claims, an efficiency of plating (EOP) test was also performed (20). Briefly, 100 μL of appropriately diluted phage lysate was mixed with 5 mL of 0.75% LB soft agar containing 100 μL of an overnight culture of the host strain CRPA190, and then overlaid on 1.5% LB agar plates. Following incubation for 12 h at 37°C, plaques were counted, and phage titer was determined. EOP was calculated as the ratio of the phage titer obtained on the test strain to that obtained on CRPA190. EOPs are reported as a percentage and represent the average EOP ± standard deviation for three biological replicates.

### Phage optimal multiplicity of infection (MOI)

The method of optimal multiplicity of infection was performed as described previously with slight changes (19, 21). The host bacterial solution was cultured until reaching the logarithmic phase. Phage dilutions were mixed with an equal volume of host bacterial solution at different MOI values (10, 1, 0.1, 0.01, and 0.001). The mixture was inoculated into sterilized LB liquid medium and incubated at 37°C for 6 h. Subsequently, the mixture was centrifuged at 5,000 × *g* for 10 min. Afterward, the supernatant was filtered through a 0.22 μm filter to remove bacteria, followed by determining the phage titer. The phage titers under different MOI ratios were recorded to determine the optimal MOI. The experiment was repeated in triplicate, and the mean ± standard deviation value was calculated.

### Adsorption of bacteriophage

Overnight-incubated CRPA190 bacteria were inoculated into 10 mL of LB broth and incubated at 37°C with shaking at 200 rpm for 12 h. Next, the bacteriophage CRPAP1904 was mixed with the strain CRPA190 to reach an MOI of 0.01 and incubated at room temperature for 10 min to facilitate phage adsorption. Samples of 500 μL mixture were taken at intervals of 1 min, followed by centrifugation at 12,000 × *g* at 4°C for 2 min. After centrifugation, 100 μL supernatants of the samples were aspirated into 900 μL saline to conduct serial dilutions and plated on double-layer agar. Phage plaques were counted after incubation at 37°C for 12 h.

### One-step growth curve experiment

The one-step growth curve experiment was conducted according to a previously described method with necessary modifications based on preliminary experiments (22). Phage CRPAP1904 was mixed with host strain CRPA190 (10^8^ CFU/mL) at the optimal MOI and incubated at 37°C for 10 min to allow adsorption. Subsequently, the mixture was centrifuged at 10,000 ×* g* for 5 min. The pellet was resuspended in pre-equilibrated LB liquid medium and incubated at 37°C with shaking at 220 rpm for 120 min. The phage titer in the solution was measured at 10-min intervals for 120 min using the double-layer agar plate method. The experiment was repeated in triplicate. The latent period is defined as the time interval between phage adsorption and the onset of the first lysis; the burst phase refers to the stage of active phage release after completing replication, characterized by a rapid increase in extracellular phage titer; and the burst size is defined as the ratio of the number of progeny phages to the number of initially infected host cells.

### Thermal and pH stability assays

To evaluate the physicochemical stability of phage CRPAP1904, its activity retention under various environmental conditions was examined (22, 23). Briefly, 1 mL of phage suspension (1.0  ×  10^8^ PFU/mL) was placed at −20°C, 4°C, 25°C, 37°C, 40°C, 50°C, 60°C, 70°C, and 80°C, respectively. Following 1 h of incubation, the phage concentration was then examined using double-agar overlay plate assay. To test the stability of phages at various pH values, the pH of the LB was adjusted to pH 3–12. One hundred of the phage suspension (1.0 × 10^9^ PFU/mL) were added to 900 μL of pH-adjusted media. Following incubation at 37 °C for 1 h, the phage concentration was then examined using double-agar overlay plate assay. All tests were performed in triplicate.

### The antibacterial ability of bacteriophage CRPAP1904 *in vitro*

Single colonies of CRPA190 were picked and incubated overnight at 37°C and 200 rpm. After inoculation in 40 mL of fresh LB broth, the suspension was incubated to the early logarithm. Subsequently, the phage was treated against grown CRPA190 at different MOIs (100, 10, 1, 0.1, and 0.01) for 20 h. The mixture was then incubated at 37°C with shaking (220 rpm). Bacterial density (OD_620_) was recorded at 2-h intervals for 20 h. A culture of host cells without the lytic phage was used as a positive control. Assays were performed in triplicate individually, and data were expressed as the mean ± standard deviation.

### Surviving bacteria re-exposed to phage CRPAP1904

To assess the emergence of phage-resistant mutants, the host bacterium CRPA190 (10^8^ CFU/mL) was incubated with its respective phage CRPAP1904 at an MOI of 100. In parallel, a phage-free control was prepared by incubating CRPA190 with LB broth. Both the phage-bacteria mixture and the control were incubated at 37°C for 12 h, after which 100 µL of each culture was spotted onto Blood Agar (BA) plates and incubated for a further 12 h at 37°C. All experiments were repeated three times. To test for phage resistance, three surviving bacterial clones (CRPA190-1, CRPA190-2, and CRPA190-3) were individually selected from the phage-treated plates. Following overnight culture, each clone, along with the parental strain CRPA190, was mixed with 5 mL of soft 0.75% LB agar and poured onto 1.5% LB agar plates, respectively. After solidification, 10 μL of purified phage CRPAP1904 suspension (10^10^ PFU/mL) was spotted onto double-layer agar plates. After adsorption of phage suspension, plates were incubated overnight at 37°C. The formation of plaques on the bacterial lawns of those strains was observed. The experiment was independently repeated three times. An EOP test was also performed. Briefly, 100 μL of appropriately diluted phage lysate was mixed with 5 mL of 0.75% LB soft agar containing 100 μL of an overnight bacterial culture (strains CRPA190, CRPA190-1, CRPA190-2, or CRPA190-3). The mixture was then overlaid on 1.5% LB agar plates. After 12 h of incubation at 37°C, plaques were counted to determine the phage titer. EOP was calculated as the ratio of the phage titer obtained on the test strain to that obtained on CRPA190. All experiments were performed with three biological replicates, and EOPs are reported as percentages.

### Genome sequencing and bioinformatics analysis

Phage DNA was extracted using the Ezup Column Virus DNA Purification Kit (Sangon Biotech, China) following the manufacturer’s protocol. The extracted genomic DNA was sequenced with the Illumina HiSeq 2500 platform (Sangon Biotech). To ensure high-quality reads for downstream analysis, the raw sequencing data underwent quality assessment using FastQC (https://www.bioinformatics.babraham.ac.uk/projects/fastqc/), followed by adapter trimming and quality filtering using Trimmomatic (https://github.com/usadellab/Trimmomatic) (24). High-quality reads were assembled using the denovo assembler SPAdes (https://ablab.github.io/spades/). The assembled genome was annotated using Prokka (https://github.com/tseemann/prokka) (25). Possible conserved domains within the protein sequences were identified using the Conserved Domains Search database (https://www.ncbi.nlm.nih.gov/cdd). Prediction of tRNAs was conducted using tRNAscan-SE 2.0 (26) and RNAmmer (v1.2) (27). All annotated genes were compared against the comprehensive antibiotic resistance database (CARD) (https://card.mcmaster.ca/) and the virulence factor database (VFDB) (http://www.mgc.ac.cn/VFs/) (28, 29). A map of the phage genome was generated using Proksee (https://proksee.ca/) (30). Based on the sequences of the terminase large subunit, phylogenetic analyses were performed with MEGA 12.0 by the neighbor-joining method with 1,000 bootstrap replicates (31). Based on the whole genome, the phylogenetic analysis of phage was performed using VICTOR (https://ggdc.dsmz.de/victor.php#) (32). Phage homology calculations were performed using VIRIDIC (https://rhea.icbm.uni-oldenburg.de/viridic/) (33). A comparative analysis of the phage gene with its closest relative was performed using Easyfig software 2.1 at the DNA level and CoreGenes5.0 with a default parameter (https://coregenes.ngrok.io/) (34, 35).

### Statistical analysis

Data were presented as mean values with standard deviations. Statistical parameters were calculated using GraphPad Prism 8.0 software. Statistical analysis was performed via one-way ANOVA analysis of variance. All experiments were conducted blindly and independently repeated under the same conditions. Statistical significance was defined as a *P* value of <0.05.

## RESULTS

### Phage isolation and morphological analysis

**Fig 1:**
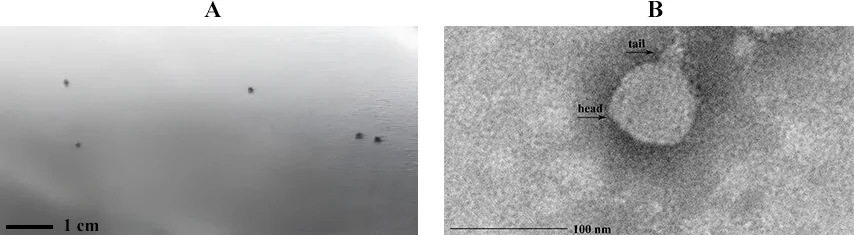
Morphological characterization of phage CRPAP1904. (**A**) Image of the clear plaques formed by the phage on a lawn of the host strain CRPA190. (**B**) Transmission electron micrograph of the phage CRPAP1904 (scale bar, 100 nm).

A novel phage was successfully isolated from a hospital sewage sample using CRPA190 as the host bacterium. On double-layer agar plates, the bacteriophage CRPAP1904 formed clear plaques with sharp edges (1.2 ± 0.3 mm, *n* = 5) (Fig. 1A). TEM showed that bacteriophage CRPAP1904 has a short tail (length 20 ± 2.4 nm, *n* = 5) and a relatively isometric head (70 ± 1.5 nm, *n* = 5) (Fig. 1B). Based on the morphological characteristics and the 2025 ICTV classification criteria (International Committee on Taxonomy of Viruses), the phage CRPAP1904 was classified within the class *Caudoviricetes*.

### Host range

Phage CRPAP1904 was tested against a panel of *P. aeruginosa* strains (Table S1), which were previously isolated from clinical samples, including tracheal aspirate, urine, wound secretion, sputum,ascites, and bronchoalveolar lavage fluid, collected between 2020 and 2023 from eight different hospital wards. The antibiotic susceptibility profiles indicated that all these strains were carbapenem-resistant *Pseudomonas aeruginosa*. The results of the spot test (Table 1) show that phage displayed lytic activity against 20 *P. aeruginosa* strains. To further explore the ability of phage CRPAP1904 to infect these strains, we performed an efficiency of plating (EOP) experiment (Table 1). Using strain CRPA 190 as reference (EOP of 100%), different EOPs among the sensitive strains were observed, and eight strains displayed high EOPs (>50%).

**TABLE 1:** Host range of phage CRPAP1904 by spot tests and efficiency of plating (EOP)^*a*^^,^^*b*^

Host strain	Source	Spot test	EOP (%)
190	Tracheal aspirate	+	100
5	Urine	+	0.04 ± 0.03
9	Wound secretion	+	0.03 ± 0.04
14	Sputum	−	0
18	Tracheal aspirate	+	0.05 ± 0.03
23	Sputum	+	0.02 ± 0.01
36	Sputum	+	15 ± 6.4
59	Tracheal aspirate	+	17 ± 4.9
92	Ascites	+	0.1 ± 0.05
101	BALF	+	9 ± 1.7
105	BALF	+	97 ± 4.8
109	Tracheal aspirate	+	94 ± 5.8
110	BALF	−	0
114	Tracheal aspirate	+	67 ± 6.7
115	BALF	−	0
119	BALF	+	0.05 ± 0.02
123	Tracheal aspirate	+	54 ± 4.0
124	Sputum	+	79 ± 4.2
128	BALF	+	15 ± 5.9
132	BALF	+	93 ± 6.2
137	Sputum	+	42 ± 2.2
141	Sputum	+	31 ± 7.0
146	Sputum	−	0
150	BALF	+	95 ± 4.2

^*a*^ +, clear zone of inhibition; −, no sensitivity to phage. BALF, bronchoalveolar lavage fluid.

^*b*^ EOP assay was compared with the parental isolation host (*P. aeruginosa* ST1971 CRPA190). Results represent the average EOP ± standard deviation for three biological replicates.

### Biological characterization of the bacteriophage CRPAP1904

**Fig 2:**
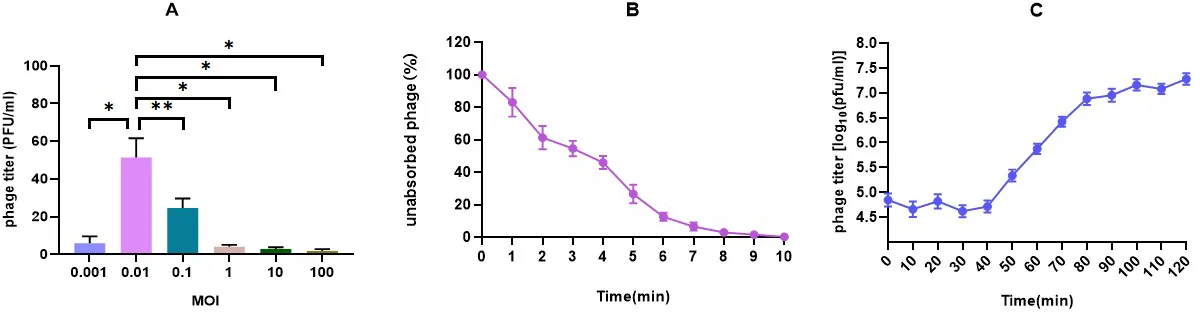
(**A**) The optimal multiplicity of infection for the phage CRPAP1904. The bacteriophage CRPAP1904 was mixed with a CRPA190 suspension at the different MOIs, and after 6 h of incubation at 37°C, the optimal MOI was determined via the double-layer agar plaque assay. (**B**) Adsorption curve of the bacteriophage CRPAP1904. The bacteriophage CRPAP1904 was mixed with CRPA190 to reach the optimal MOI of 0.01. While the mixture was incubated with shaking, samples were taken every minute. (**C**) One-step growth curve of the bacteriophage CRPAP1904. The bacteriophage CRPAP1904 was mixed with CRPA190 at an MOI of 0.01. After 10 min of adsorption, the suspension was incubated with shaking for 120 min, after which samples were collected and diluted for plaque counting at the indicated intervals. The data are presented as the means ± SD. Statistical analysis was performed via one-way ANOVA analysis of variance following Dunnett’s multiple comparisons test. Error bars represent the SD. *P < 0.0001; **P < 0.001.

To determine the optimal multiplicity of infection (MOI) of the bacteriophage CRPAP1904 for CRPA190, the bacteriophage was mixed with CRPA190 (10^8^ CFU/mL) at different MOIs. The phage titer reached a maximum when the mixture was incubated at an MOI of 0.01 at 37°C for 6 h (*P* < 0.05), suggesting that the optimal MOI of the bacteriophage CRPA1904 was 0.01 (Fig. 2A). Adsorption tests revealed that 99% of the phage particles could successfully adsorb to the host cells after 10 min of incubation (Fig. 2B). For the one-step growth curve at the optimal MOI of 0.01, the phage titer remained stable, with no significant changes during the first 40 min post-infection (Fig. 2C). However, from 40 to 80 min, the titer increased rapidly, indicating active phage replication and release. The latent period was approximately 40 minutes, while the lysis phase lasted 40 min, with an average burst size of approximately 109 PFU/cell (1 × 10^6.886^/1 × 10^4.848^).

### Stability under physicochemical conditions

**Fig 3:**
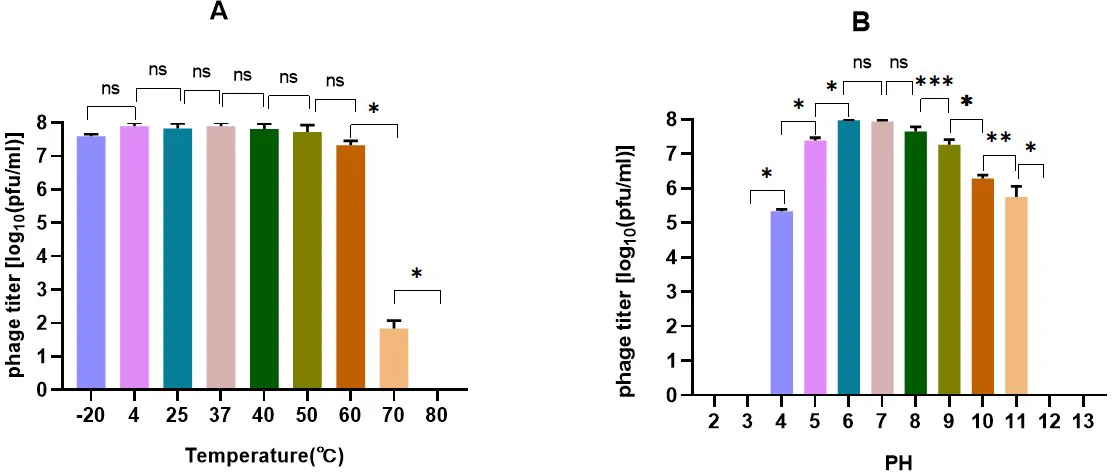
(**A**) Thermal stability of phage CRPAP1904. Phage was incubated for 1 h under different temperatures. (**B**) pH stability of phage CRPAP1904. Phage was incubated for 1 h under different pH values. The data are presented as the means ± SD. Statistical analysis was performed via one-way ANOVA analysis of variance following Tukey’s multiple comparisons test. Error bars represent the SD. *, *P* < 0.0001; **, *P* < 0.001; ***, *P* < 0.05; ns, *P* > 0.05 not significant.

Measuring the tolerance of the phage to environmental conditions is a critical step for assessing its potential applications. In Fig. 3A, thermostability testing revealed no significant change in phage titer after 1 h of incubation at temperatures ranging from −20°C to 60°C (*P* > 0.05), whereas a significant reduction in phage titer was observed at 70°C (*P* < 0.05). In Fig. 3B, the bacteriophage exhibited activity across a range of pH values (pH = 4–11). CRPAP1904 maintained relatively high infectivity at pH 4, 5, 9, 10, and 11, although the titer was significantly reduced compared to that at pH 6–8 (*P* < 0.05). CRPAP1904 displayed a stability to pH ranging from 6 to 8 (*P* > 0.05), with complete inactivation at extreme pH values of 2, 3, 12, and 13. Phage CRPAP1904 presents high physicochemical stability against environmental influences, making it a promising biocontrol agent for bacterial therapy.

### The antibacterial ability of bacteriophage CRPAP1904

**Fig 4:**
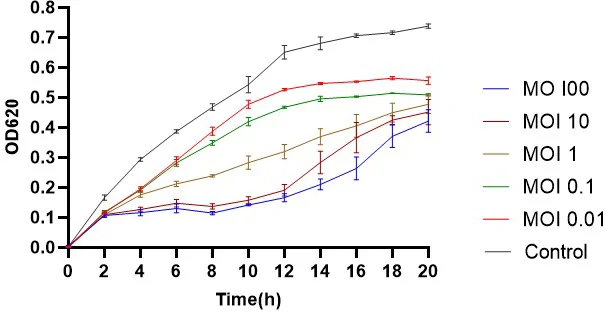
Bacteriophage kill-curves of CRPA190 in response to infection by phage CRPAP1904. The host was infected with the CRPAP1904 at different MOIs. The positive control (only bacteria) is represented with black color. All samples were analyzed in triplicate. Results are expressed as mean values ± SD. Error bars represent the SD.

To further assess its application potential, the phage was used to inhibit the growth of CRPA190 in LB broth. Following the addition of phage CRPAP1904, the growth of the bacterial host was inhibited at MOI values ranging from 0.01 to 100, compared with the control group (Fig. 4). Our results revealed that the impact of phage CRPAP1904 on bacterial growth is concentration dependent. When the host bacteria were infected at low MOIs (MOI of 0.01, 0.1, or 1), a slight reduction of bacterial growth was observed. On the other hand, when the host bacteria were infected at high MOIs (MOI of 10 or 100), the growth of the bacteria CRPA190 was dramatically inhibited, with a substantially decreased OD_620_ value within the first 12 h. However, after 12 h, a gradual increase in bacterial concentration was observed in the MOI 100 treatment group.

### Emergence of bacteriophage CRPAP1904 resistance

**Fig 5:**
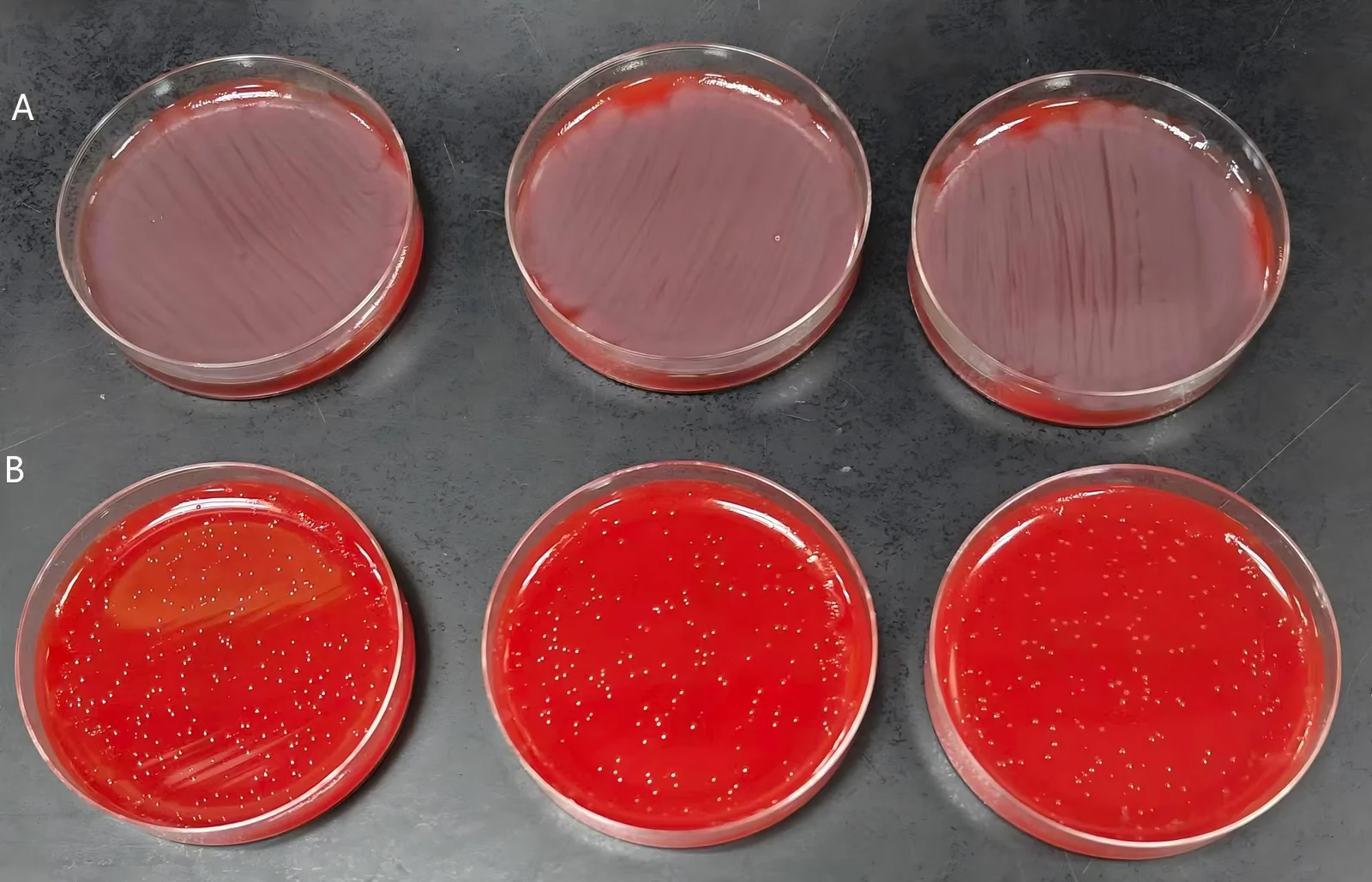
Screening for phage CRPAP1904 resistant mutants. (**A**) Control group (CRPA190 in LB broth, 12 h) shows confluent bacterial lawn on blood agar plates. (**B**) Treatment group (CRPA190 with phage CRPAP1904, MOI = 100, 12 h) exhibits sparse colony formation on blood agar plates.

After 12 h of incubation, the control group (CRPA190 cultured in LB broth without phage treatment) formed a confluent bacterial lawn (Fig. 5A). In contrast, co-incubation of CRPA190 with its specific phage CRPAP1904 at an MOI of 100 for 12 h resulted in only sparse colony formation (Fig. 5B). Then, the surviving bacteria from the MOI 100 treatment group were re-exposed to the phage CRPAP1904. A clear zone was observed on the bacterial lawns of the control strain CRPA190, whereas no such zone formed on the lawns of the surviving strains (CRPA190-1, CRPA190-2, and CRPA190-3) (Table 2). The absence of lysis in the surviving strains confirms their resistance to phage CRPAP1904. Furthermore, using strain CRPA190 as the reference (assigned an EOP of 100%), the selected surviving bacterial variants (CRPA190-1, CRPA190-2, and CRPA190-3) all exhibited an EOP of 0%, confirming the emergence of phage resistance.

**TABLE 2:** Assessment of phage CRPAP1904 resistance emergence by spot test and efficiency of plating (EOP) assay^*a*^

Host strain	Phage	Spot test	EOP (%)
190	CRPAP1904	+	100
190-1	CRPAP1904	−	0
190-2	CRPAP1904	−	0
190-3	CRPAP1904	−	0

^*a*^ +, clear zone of inhibition; −, no sensitivity to phage.

### Genomic analysis of phage CRPAP1904

#### Genome composition and gene function annotation

**Fig 6:**
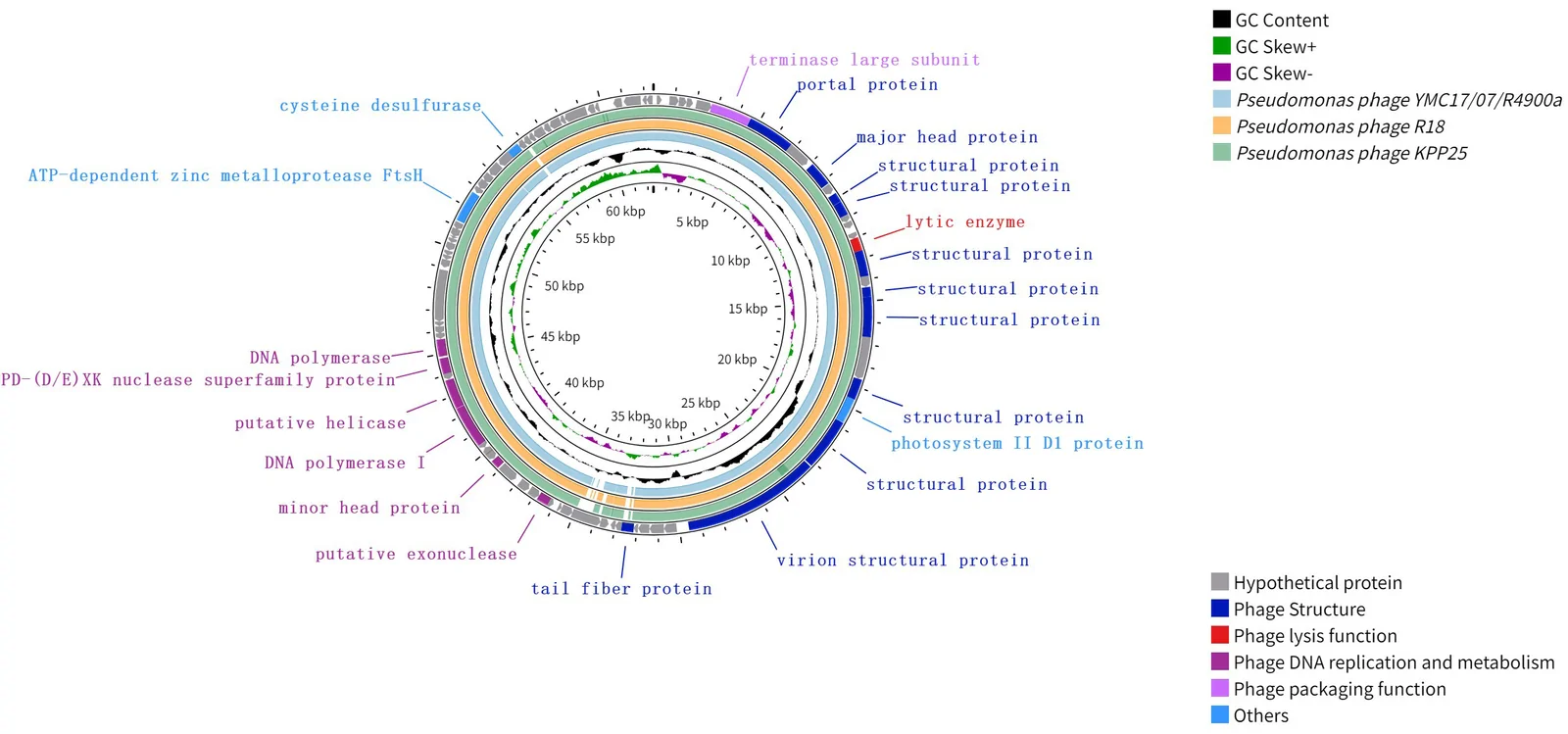
Circular genome map of phage CRPAP1904. The innermost ring represents the GC skew while the central ring (black) shows the GC content. BLAST alignment was performed for sequencing similarity comparison versus *Pseudomonas phage YMC17/07/R4900a* (baby blue ring), *Pseudomonas phage R18* (yellow ring), and *Pseudomonas phage KPP25* phage (green ring). The outermost circle displays the coding genes (CDS) for CRPAP1904 phage with predicted functions as labels. The coloration of the CDS corresponds to their general functions as indicated in the legend.

The complete genome sequence of the phage has been uploaded to the NCBI GenBank database under accession number PQ741025. The genomes consisted of 62,430 bp double-stranded DNA, with a G + C content of 60.4%. CRPAP1904 contains 83 predicted open reading frames (ORFs), and no tRNAs were detected in the CRPAP1904 genome. Twenty-two of the ORFs were assigned predicted functions, and 61 out of 83 predicted ORFs were assigned to hypothetical proteins with unknown functions, possibly attributed to the limitations of the database used for annotation and the homogeneous number of phages. The annotated genes from the phage were categorized into five groups: structure, lysis, packaging, DNA replication and metabolism, and others (Fig. 6). Ten conserved domains were identified in the protein sequences involved in virion DNA replication, transcriptional regulation, lysis module, and structural protein formation (Table 3). Analysis against the CARD and VFDB databases did not detect genes homologous to known antibiotic resistance genes or known virulence factors in the CRPAP1904 genome.

**TABLE 3:** Functional genes analysis of the CRPAP1904 genome and their conserved domains^*a*^

Gene	Function	Accession	Conserved domain name	Description
6	Terminase large subunit	COG5410	COG5410	Often fused with C-terminal phage terminase domain
7	Portal protein	–	–	–
9	Major head protein	pfam13252	DUF4043 super family	Proteins in this family are typically between 369 and 424 amino acids in length
11	Structural protein	–	–	–
12	Structural protein	–	–	–
16	Lytic enzyme	COG3179	GH19	Releases phage progeny by degrading the peptidoglycan of host cell walls
17	Structural protein	–	–	–
19	Structural protein	–	–	–
20	Structural protein	–	–	–
22	Structural protein	–	–	–
23	Photosystem II D1	cd15482	Sialidase_non-viral	Sialidase that removes sialic acid residues from glycoconjugates
24	Structural protein	–	–	–
25	Virion structural protein	PHA01972	PHA01972 super family	Virion structural protein
30	Tail fiber protein	–	–	–
38	Putative exonuclease	pfam16473	Rv2179c-like	3′−5′ exonuclease catalyzes the excision of nucleoside monophosphates at the DNA or RNA termini in the 3′−5′ direction; belongs to the DnaQ-like (or DEDD) 3′−5′ exonuclease superfamily
42	Minor head protein	–	–	–
46	DNA polymerase	cd06444	DNA_pol_A super family	DNA polymerase A family protein functions primarily to fill DNA gaps that arise during DNA repair, recombination and replication
47	Putative helicase	COG0553	HepA super family	DEAD/DEAH box containing ATP-dependent helicase catalyzes the unwinding of DNA or RNA
49	PD-(D/E) XK nuclease superfamily protein	COG2887	Slr0479	RecB family exonuclease similar to exodeoxyribonuclease V subunit beta (RecB), a component of the heterotrimeric RecBCD helicase/nuclease complex that is essential for double-strand DNA break repair and recombination
50	DNA polymerase	–	–	–
63	Cell division protein FtsH	COG1222	RPT1 super family	ATP-binding protein with an AAA (ATPases Associated with various cellular Activities) domain may function as an ATPase
69	Cysteine desulfurase	–	–	–

^*a*^ –, no identified.

#### Phylogenetic analysis of phage CRPAP1904

**Fig 7:**
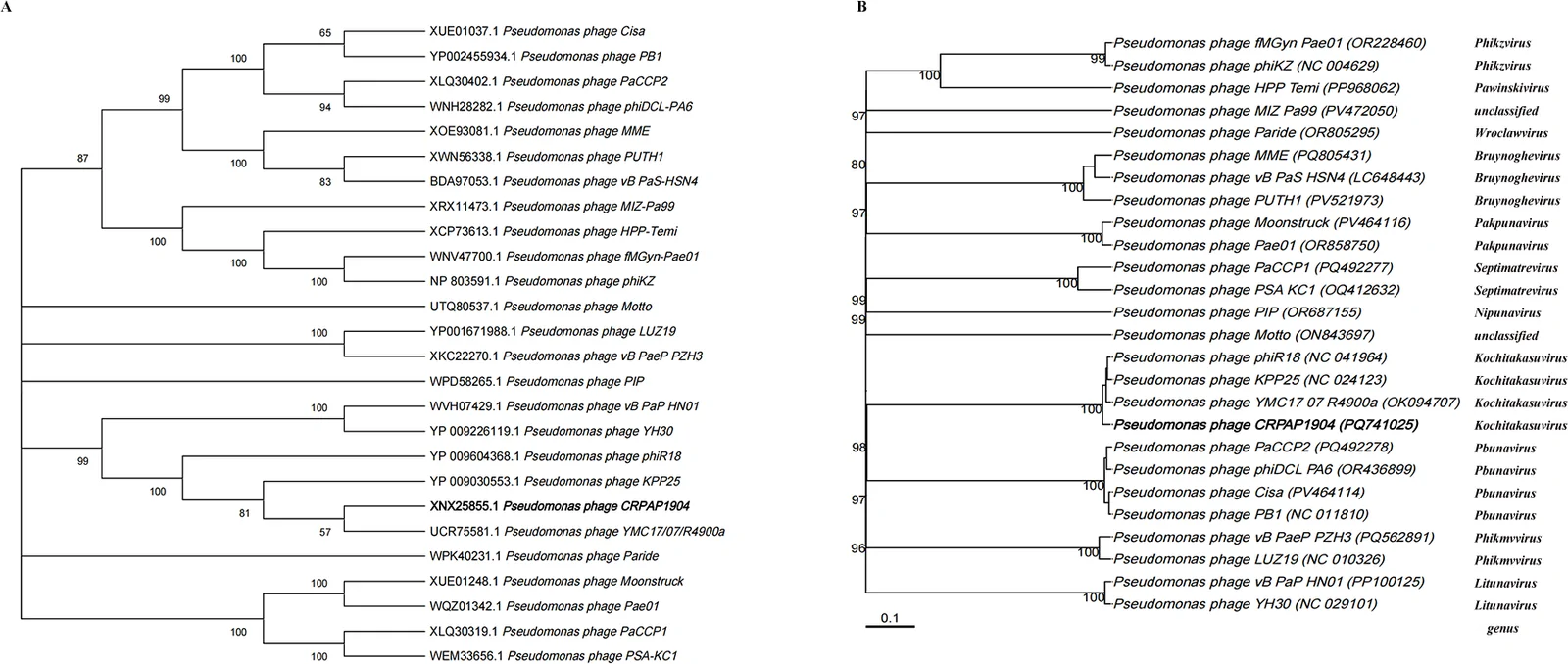
Phylogenetic analysis. (**A**) Phylogenetic tree using terminase large subunit. The phylogenetic trees were constructed using the neighbor-joining (NJ) method and evaluated with 1,000 bootstrap replicates using MEGA 12 software. (**B**) Phylogenetic tree using whole-genome VICTOR analysis.

The phylogenetic trees were constructed based on the terminase large subunit and the whole genome, respectively. In the terminase large subunit-based phylogeny, phage CRPAP1904 clustered with *Pseudomonas* phages KPP25 (NC_024123.1) (36), *Pseudomonas* phage phiR18 (NC_041964.1) (37), and *Pseudomonas* phage YMC17/07/R4900a (OK094707.1), forming a distinct clade separate from phages of other genera (Fig. 7A). Furthermore, in Fig. 7B, CRPAP1904 forms a distinct phylogenetic clade, separate from all other members of the genus *Kochitakasuvirus*. The whole-genome phylogenetic analysis supports that CRPAP1904 represents a novel species within the genus *Kochitakasuvirus*.

#### Comparative genomic analysis

**Fig 8:**
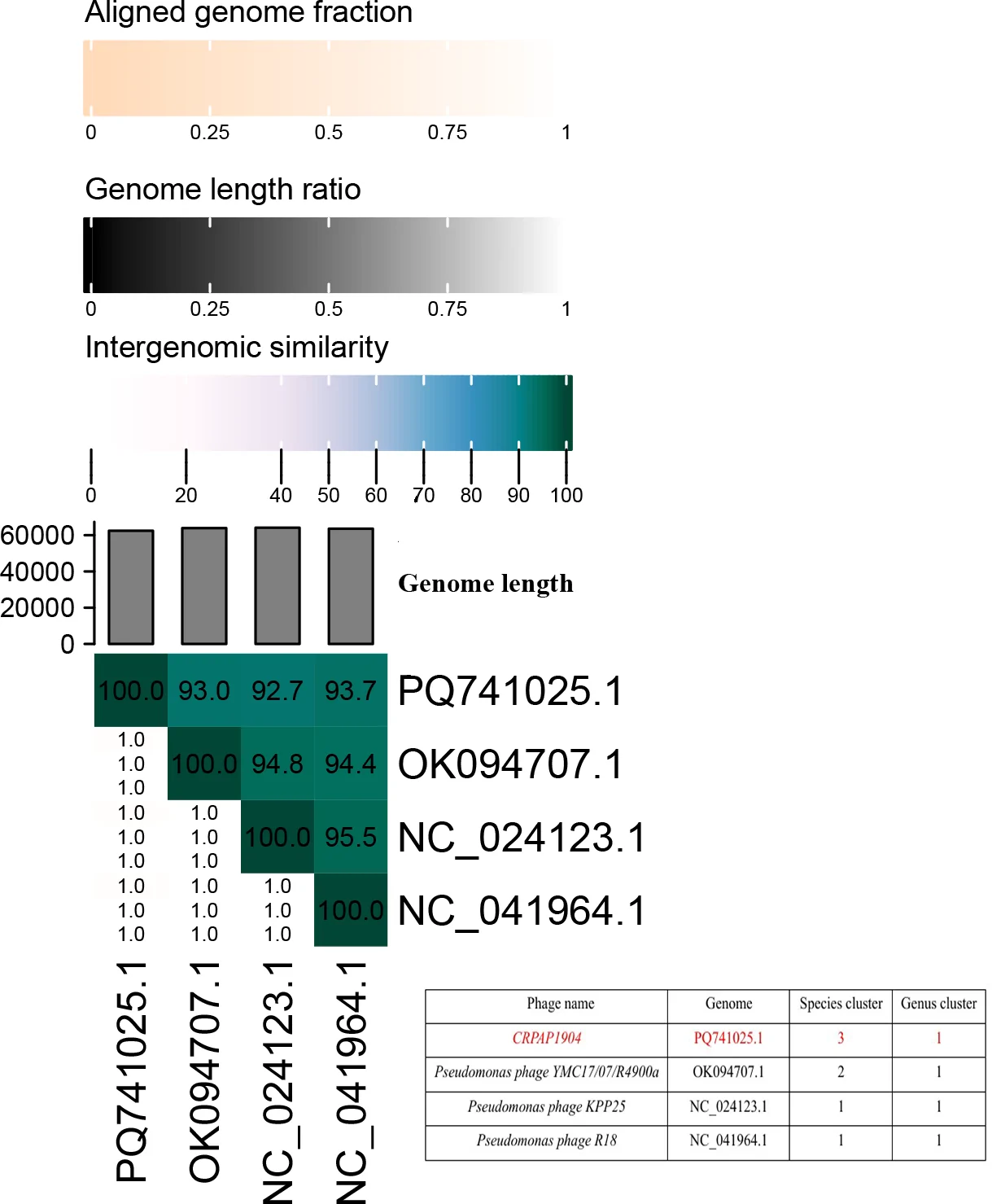
VIRIDIC heatmap based on intergenomic similarities between CRPAP1904 and other *Kochitakasuvirus* phages. The number in the chart represents the similarity percentage. The horizontal and vertical coordinates indicate the corresponding phage GenBank number. Aligned genome fraction: the proportion of a genome that can be successfully aligned to another genome during a pairwise comparison. Genome length ratio: smaller genome length/bigger genome length.

**Fig 9:**
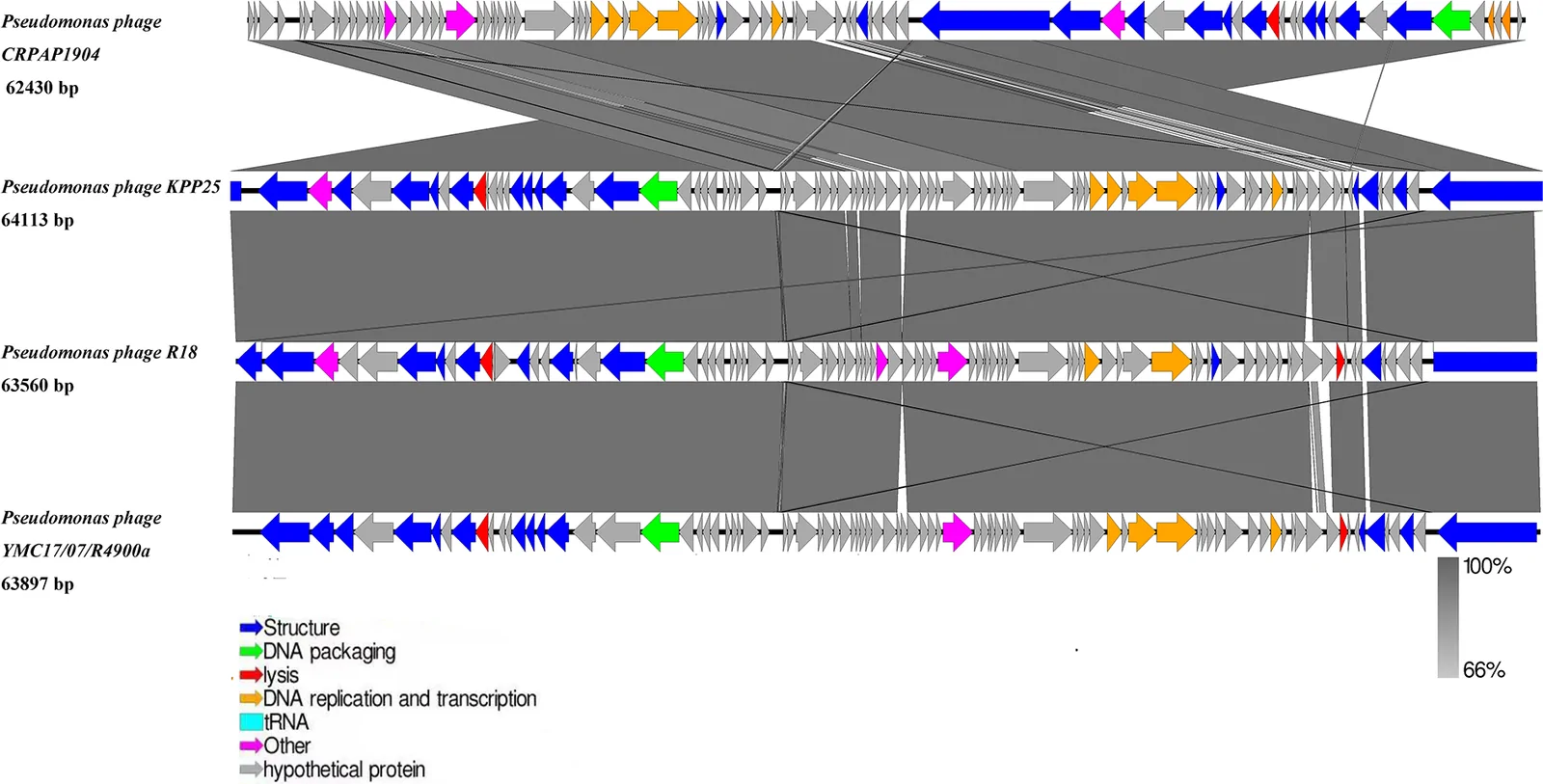
Genome comparison of CRPAP1904 with closely related members of the *Kochitakasuvirus* genus. The gray shading indicates sequence similarities between the genomes. The predicted functions of proteins are indicated by different colors. blackish green arrows represent ORFs. The phage genomes were compared by Easyfig 2.1.

VIRIDIC was used to calculate the intergenomic similarities between phage CRPAP1904 and other known closely related *P. aeruginosa* phages (Fig. 8). Intergenomic similarity heatmap showed pairwise sequence identity between phage CRPAP1904 and other related *Kochitakasuvirus* was 92.7–93.7%, indicating that CRPAP1904 is a new member of the *Kochitakasuvirus* genus within *Caudoviricete*. Strong similarities to *Pseudomonas phage KPP25, Pseudomonas* phage phiR18, and *Pseudomonas* phage YMC17/07/R4900a at the nucleotide level and their genomic organization were detected by genome comparison visualized using Easyfig 2.1 (Fig. 9). CoreGenes5.0 indicates that CRPAP1904 shares 80 homologous genes in common with *Pseudomonas* phage YMC17/07/R4900a*,* mostly composed of genes of unknown function. CRPAP1904, *Pseudomonas* phage YMC17/07/R4900a, *Pseudomonas* phage KPP25, and *Pseudomonas* phage phiR18 feature high homology in sequences coding proteins, which are thought to be involved in the virion structure and packaging.

## DISCUSSION

The rise of multidrug-resistant and extensively drug-resistant strains has further compounded the global burden of *P. aeruginosa* infections (38). Our previous work identified CRPA190 as an ST1971 clone. This isolate was characterized by extensive drug resistance to most conventional antibiotics, a profile that severely limited therapeutic options (10). So new treatment options are needed to address the pathogen. Bacteriophages, as the most abundant biological entities in the environment, utilize host bacteria as replication factories to facilitate their own propagation while simultaneously regulating bacterial populations within specific microenvironments (39, 40). These natural bacterial predators are primarily classified into two distinct categories based on their replication strategies: temperate (lysogenic) bacteriophages and virulent (lytic) bacteriophages. Lytic phages are characterized by their immediate replication within and subsequent lysis of host bacteria. This lytic cycle results in the production of numerous progeny virions and host cell destruction, making these phages particularly valuable for therapeutic applications (23). The unique properties of lytic phages have inspired innovative approaches to combat refractory multidrug-resistant pathogen infections (41). To treat drug-resistant bacterial infections more effectively, it is essential to build and optimize phage libraries to find phages targeting specific drug-resistant bacteria (42). Thus, expanding our knowledge of bacteriophage biology will directly translate into broader therapeutic applications for combating resistant infections.

This study reports the isolation and characterization of a novel lytic bacteriophage CRPAP1904 against an extremely drug-resistant *Pseudomonas aeruginosa* ST1971 strain CRPA190. Transmission electron microscopy confirmed that CRPAP1904 possesses characteristic *Caudoviricetes* morphology, featuring an icosahedral capsid and non-contractile tail assembly. Because the genes encoded on the phage CRPAP1904 genome lack sequence homology to known integrases, it was thought that phage CRPAP1904 is a lytic phage. Bioinformatic analysis revealed that phage CRPAP1904 does not contain any tRNA genes, which suggests that the virus is highly dependent on the host translation machinery for its protein synthesis (43). Genome analysis identified 22 ORFs with known functions, including most of the functional genes essential for phage reproduction. Of these, 10 conserved domains were identified in the protein sequences. However, 61 ORFs did not correspond to any known functions. Further functional studies on these ORFs will deepen the understanding of phage CRPAP1904.

Through genome sequencing and comparative genomic analyses, coupled with phylogenetic reconstruction based on the terminase large subunit gene and the whole genome, we demonstrate that phage CRPA1904 belongs to the genus *Kochitakasuvirus* within the *Caudoviricetes* class. The ICTV guidelines recommend a genome sequence identity threshold of 95% for the demarcation of main species within archaeal viruses (44). Notably, the intergenomic similarity heatmap revealed that CRPAP1904 shares only 92.7–93.7% genome sequence identity with its closest relatives, including *Pseudomonas* phage YMC17/07/R4900a, *Pseudomonas* phage KPP25, and *Pseudomonas* phage phiR18. This level of sequence identity falls below the 95% species demarcation threshold recommended by ICTV, thereby supporting the classification of CRPAP1904 as a novel phage species.

The phage genome encodes a suite of enzymes, including exonuclease (ORF38), DNA polymerase (ORF46 and ORF50), and helicase (ORF47), which collectively enable the phage to disrupt host DNA integrity, execute DNA replication and recombination, and complete independent transcriptional processes. The exonuclease protein contains a conserved domain, which may play a role in the final step of host DNA degradation, by scavenging DNA into mononucleotides. DNA polymerase protein functions primarily to fill DNA gaps that arise during DNA repair, recombination, and replication. Helicases play a crucial role in viral genome transcription, replication, recombination, and repair. The lysis module plays a crucial role in the host cell disruption and the release of progeny virions (45). Further analysis of the functional gene encoding lytic enzyme (ORF16) may elucidate the phage’s lytic mechanism, providing new insights into how the phage precisely degrades bacterial cell wall and may have significance for the development of new antibacterial therapies to combat MDR-PA.

Our host range analysis results demonstrate that phage CRPAP1904 can specifically lyse MDR-PA clinical isolates, thereby expanding the repertoire of phages available to combat chronic *P. aeruginosa* infections. The therapeutic potential of phages in treating bacterial infections is largely determined by their biological characteristics, with optimal clinical candidates typically exhibiting three key traits: a high burst size, a short latent period, and strong lytic activity (23). Our results indicate that the phage exhibits a 40 min latent period and the lysis phase lasted 40 min, with an average burst size of approximately 109 PFU/cell. The phage can rapidly complete the processes of infection, replication, and progeny release. The bacteriophage CRPAP1904 exhibited high burst sizes and strong lytic ability. These detailed characterizations herein provide the necessary framework for further clinical usage. Bacteriophages with broad lytic activity against antibiotic-resistant bacteria represent excellent candidates for novel therapeutic agents. Their application is envisioned either as a monotherapy for resistant infections or as part of a combination therapy regimen (46).

The stability of bacteriophages in solution is a critical characteristic for their therapeutic use (47). Phage CRPA1904 maintains a high titer at temperatures below 50°C and remains active in the pH range of 4–11, indicating its broad tolerance to heat and pH. Most importantly, the genome of phage CRPA1904 appears to lack antibiotic resistance and virulence genes, which makes it more suitable for phage therapy. Although these characteristics are encouraging, further experimental validation is still required to comprehensively evaluate its safety for humans and the environment. With additional assessment, it has the potential to serve as an antibiotic alternative for hospital-acquired infections and fulfill its promise as an antimicrobial agent.

Phages have different ideal MOIs. The optimal MOI for this phage was determined to be 0.01. The highest inhibitory capacity was observed in the MOI 100, and the inhibitory effect was MOI-dependent. The bacteriophage CRPAP1904 exhibited the highest inhibitory activity within the first 12 h at an MOI of 100. However, bacterial growth curves under MOI 100 conditions revealed that OD_620_ values began to increase after approximately 12 h, suggesting the potential emergence and proliferation of phage-resistant mutants. To confirm the existence of phage-resistant variants, the surviving bacteria from the MOI 100 treatment were re-exposed to the phage CRPAP1904. The results showed that the surviving bacteria exhibited resistance to CRPAP1904. This finding suggested that the phage-resistant mutants were selected by CRPAP1904. In many cases, there is a gradual increase in the numbers of phage-resistant bacteria. For instance, the phage-resistant variants were isolated from planktonic cultures of *P. aeruginosa* PAO1 infected with phage LUZ19, phiIBB-PAA2, and vB_PaeM_CEB_DP1, respectively (48). In laboratory environments, the selective pressure that is exerted by phages is shown to act as a trigger for host evolution and a factor that influences host (49). Phages, especially virulent phages, have greatly contributed to bacterial evolution owing to their persistent threat, and a diverse set of phage defense systems for withstanding phage predation have been developed in bacteria during the coevolutionary process with phages (50–53). To survive and/or escape phage predation, bacteria have evolved and acquired sets of resistance mechanisms, including adsorption-blocking systems that prevent phage adsorption to cell receptors, superinfection exclusion (Sie) systems that prevent phage DNA entry into the host cell, modification systems that prevent phage infection by cleaving phage genomic DNA, and CRISPR–Cas systems that confer immunity against incoming foreign DNA, and bacteria even have evolved a plethora of intracellular proteins that cause abortion of the phage infection (54, 55). Previous studies revealed that there were three CRISPR arrays and two Cas clusters (CAS-TypeIF and CAS-TypeID) on the genome of the CRPA190 strain, which could be affecting phage activity (10). The presence of phages can also affect the virulence, capacity for biofilm formation, or antibiotic resistance of a pathogen, both positively and negatively (49). For instance, *P. aeruginosa* PA1 strains that are resistant to phage PAP-1 exhibit decreased virulence due to the loss of the O-antigen (56). Mutant PA1RG exhibited decreased biofilm production, suggesting a fitness cost of PA1 associated with resistance to phage PaP1 predation (57). The occurrence of phage resistance limits its sole usage to combat bacterial infection. Several strategies have been developed to address the emergence of phage resistance during the clinical application of phage therapy, such as the use of a phage cocktail that contains different types of phages and the combination of phages with other antimicrobial agents (48, 58). Some works already demonstrated synergistic effects between phages and antibiotics (59–61). In the studies reported by Oechslin, phage-resistant mutants regrew after 24 h but were prevented by the combination with ciprofloxacin. Furthermore, phage-resistant mutants emerged *in vitro* but not *in vivo* (59). Future research should focus on screening additional *P. aeruginosa* phages and developing efficient, broad-spectrum phage cocktail therapies with the aim of optimizing phage therapy regimens and providing a theoretical foundation for the prevention and control of multiple-drug-resistant *P. aeruginosa* infections.

### Conclusion

Here, we isolated and identified a phage CRPAP1904, and its physicochemical properties were extensively investigated. We sequenced the phage and found that phage CRPAP1904 belonged to the genus *Kochitakasuvirus* of the class *Caudoviricetes*. Importantly, we demonstrated that CRPAP1904 is effective in lysing host bacteria. This work expands phage resources and supports medicinal phage library development, advancing phage therapy and biocontrol applications in MDR-PA infections. Further research is needed to optimize phage therapy protocols.

## Data Availability

The genome of CRPAP1904 was deposited in the GenBank database under accession number PQ741025. The data of the study are with the corresponding author of the article and can be made available on request.

## SUPPLEMENTAL MATERIAL

Table S1 spectrum.02713-25-s0001.xlsx Detailed information and antibiotic susceptibility profiles of the test strains.

ASM does not own the copyrights to Supplemental Material that may be linked to, or accessed through, an article. The authors have granted ASM a non-exclusive, world-wide license to publish the Supplemental Material files. Please contact the corresponding author directly for reuse.
